# Preparation and Biological Activity of the Monoclonal Antibody against the Second Extracellular Loop of the Angiotensin II Type 1 Receptor

**DOI:** 10.1155/2016/1858252

**Published:** 2016-01-20

**Authors:** Mingming Wei, Chengrui Zhao, Suli Zhang, Li Wang, Huirong Liu, Xinliang Ma

**Affiliations:** ^1^Department of Physiology and Pathophysiology, School of Basic Medical Sciences, Capital Medical University, Beijing 100069, China; ^2^Beijing Key Laboratory of Metabolic Disorders Related Cardiovascular Diseases, Capital Medical University, Beijing 100069, China; ^3^Department of Physiology, Basic Medical Department, Fenyang College of Shanxi Medical University, Fenyang, Shanxi 032200, China; ^4^Department of Pathology, Shanxi Medical University, Taiyuan, Shanxi 030001, China; ^5^Department of Emergency Medicine, Thomas Jefferson University, 1025 Walnut Street, College Building, Suite 808, Philadelphia, PA 19107, USA

## Abstract

The current study was to prepare a mouse-derived antibody against the angiotensin II type 1 receptor (AT1-mAb) based on monoclonal antibody technology, to provide a foundation for research on AT1-AA-positive diseases. Balb/C mice were actively immunized with the second extracellular loop of the angiotensin II type 1 receptor (AT_1_R-ECII). Then, mouse spleen lymphocytes were fused with myeloma cells and monoclonal hybridomas that secreted AT1-mAb were generated and cultured, after which those in logarithmic-phase were injected into the abdominal cavity of mice to retrieve the ascites. Highly purified AT1-mAb was isolated from mouse ascites after injection with 1 × 10^7^ hybridomas. A greater amount of AT1-mAb was purified from mouse ascites compared to the cell supernatant of hybridomas. AT1-mAb purified from mouse ascites constricted the thoracic aorta of mice and increased the beat frequency of neonatal rat myocardial cells via the AT_1_R, identical to the effects of AT1-AA extracted from patients' sera. Murine blood pressure increased after intravenous injection of AT1-mAb via the tail vein. High purity and good biological activity of AT1-mAb can be obtained from mouse ascites after intraperitoneal injection of monoclonal hybridomas that secrete AT1-mAb. These data provide a simple tool for studying AT1-AA-positive diseases.

## 1. Introduction

Angiotensin II (Ang II) receptors are a class of G-protein-coupled receptors that exist in four subtypes: AT_1_R–AT_4_R. The angiotensin II type 1 receptor (AT_1_R) is mainly expressed in vascular smooth muscle cells (VSMCs), endothelial cells, and myocardial fibroblasts [1] and as such plays a prominent role in regulating the cardiovascular system. Ang II can activate the AT_1_R, thereby increasing vascular tension, causing vasoconstriction, and increasing the force of cardiac muscular contractions. However, excessive activation of AT_1_R can cause cardiovascular pathologies such as hypertension [2], vascular injury [3], arrhythmia [4], and myocardial hypertrophy [5].

Preeclampsia is a serious type of pregnancy-induced hypertension that clinically manifests itself in the form of high blood pressure and proteinuria after 20 weeks of pregnancy. Numerous studies have reported that excessive AT_1_R activation is an important mechanism underlying the occurrence and development of preeclampsia. Angiotensin II 1 type autoantibodies (AT1-AA) are agonists of AT_1_R that can cause excessive activation [6] by interacting with the second extracellular loop of the AT_1_R (AT_1_R-ECII) [7], thereby causing high blood pressure and proteinuria, which are the typical signs and symptoms of preeclampsia in pregnant rats. These findings suggest that AT1-AA may play an important role in the pathology of preeclampsia [8]. Therefore, evaluating the functions of AT1-AA and its underlying mechanisms and targets has become a major research focus. However, obtaining enough highly purified AT1-AA to establish animal models has been a considerable problem, as to date only limited amounts of antisera from clinical patients with preeclampsia have been isolated. To study the pathophysiological roles of AT1-AA, it is important to establish a more simple and productive method for the preparation of these autoantibodies.

In the present study, we prepared a mouse-derived antibody against the AT_1_R-ECII (AT1-mAb) using monoclonal antibody technology. Then, we identified the biological activities of AT1-mAb and compared them to AT1-AA purified from preeclamptic patients. This research is aim to find a simple and effective way to gain AT1-mAb to study AT1-AA positive disease, so as to provide basis for clinical treatment.

## 2. Materials and Methods

### 2.1. Experimental Animals and Materials

Our experiments were approved by the Institutional Animal Care and Use Committee of Capital Medical University (Beijing, China) and conformed to the Guiding Principles in the Use and Care of Animals published by the National Institutes of Health (NIH Publication number 85-23, revised 1996). Animals were provided by Vital River, License: SCXK (Beijing), 2012-0001. Before the experiments, the mice were fed* ad libitum* and maintained in 12-hour light/dark cycles. Healthy, 12-week-old Balb/C mice (*n* = 60; 45 females, 15 males; body weight, 18–20 g) were used for preparation of ascites (vehicle group: *n* = 10, hybridomas (10^7^) group: *n* = 10, females), isolated vascular ring experiment (*n* = 20; 15 females, 5 males), and experiments* in vivo* (*n* = 20; 10 females, 10 males), and 0–3-day-old newborn Wistar rats (*n* = 30; weight, 4–6 g) were used for neonatal rat cardiomyocytes beat frequency experiment. We observed these rats at least twice daily. They were given pentobarbital sodium (150 mg/kg) [9] by intraperitoneal injection (IP) to reduce anxiety for surgical anesthesia. Once the experiment was completed, all Balb/C mice were euthanized by decapitation at the guillotine (a physical method was suggested by AVMA Guidelines on Euthanasia).

### 2.2. Purification of AT1-AA from Patients' Sera

Six preeclampsia women were recruited from the Taiyuan Central Hospital (Taiyuan, Shanxi province, China) (Table 1). This investigation was conducted according to the principles expressed in the Declaration of Helsinki. This research protocol was approved by the Ethics Committee for the Protection of Human Subjects of Taiyuan Central Hospital. All patients had given written consent. Before serum was collected, 10 mL fasting blood samples were collected from these six subjects through cubital veins and stand at room temperature for 1 hour and then centrifuged at 3000 rpm for 15 min. The sera were isolated and stored at 20°C for purification of AT1-AA [10] over Protein G affinity column (Sweden).

### 2.3. Hybridoma Preparation

Peptides against the human AT_1_R-ECII antigen epitope (murine and human AT_1_R-ECII share 92% homology, Supplementary Figure 1 (see Supplementary Material available online at http://dx.doi.org/10.1155/2016/1858252)) were synthesized (I-H-R-N-V-F-F-I-E-N-T-N-I-T-V-C-A-F-H-Y-E-S-Q-N-S-T; GenBank AAB34644.1) at 98% purity. The peptides coupled to keyhole limpet hemocyanin (KLH) protein were used as immunogens, and those coupled to bovine serum albumin (BSA) were used as the control. This peptide was used as an antigen to immunize the mice to prepare sera containing high titers of polyclonal antibodies, and then the cells were fused to generate the hybridoma cell lines (Beijing B&M Biotech Co., Ltd.). In total, 40 strains of monoclonal hybridoma cell lines were generated, and the 5 strains that secreted the highest titers of AT1-mAb in the cell culture supernatant were identified by Biotin-Avidin Enzyme-Linked Immunosorbent Assay (BA-ELISA). Specific hybridomas were frozen in liquid nitrogen until use.

### 2.4. Hybridoma Culture

(1) For thawing, the hybridomas were quickly placed in a CryoTube at 37°C in a constant-temperature water bath, transferred to 10 mL Roswell Park Memorial Institute (RPMI) 1640 medium (HyClone, China), and centrifuged at 3000 rpm for 10 min at 4°C after mixing, after which the supernatant was removed. (2) For culturing, the cells were grown in RPMI supplemented with 10% fetal bovine serum (Gibco, USA). After 24 h, the supernatant was removed and fresh culture medium was added to the cells. (3) For subculturing, cells in logarithmic growth phase were cultured for about 3 days until 80–90% confluency, after which the supernatant was collected and frozen at −20°C until purification. The remaining cells were also collected and used for ascites preparation. (4) For cryopreservation and storage of cells, half-adherent cells were centrifuged at 1000 rpm for 5 min. Then, the supernatant was removed and the cells were cryopreserved in fetal bovine serum : RPMI : dimethyl sulfoxide (DMSO) at a ratio of 5 : 4 : 1.

### 2.5. Extraction of AT1-mAb from Hybridoma Culture Supernatant and Mouse Ascites

(1) Extraction of AT1-mAb from hybridoma culture supernatant: the proteins in the hybridoma culture supernatant were concentrated at a ratio of 5 : 1 and filtered using a 0.45 *μ*m filter, and the IgG of the hybridoma culture supernatant was purified over a protein G affinity column. (2) Extraction of AT1-mAb from mouse ascites: incomplete Freund's adjuvant (0.02 mL/g, Sigma, St. Louis, MO, USA) was injected into the abdominal cavity of all Balb/C mice. Three days later, 1 × 10^5^–1 × 10^9^ hybridomas suspended in 0.4 mL phosphate-buffered saline (PBS) were introduced into mouse abdominal cavity by IP injection with a 1 mL syringe. Ascites formation was observed by weighed and abdominal shape at every week, ascites fluid was collected in the second week, and the supernatant was collected after centrifugation at 1000 rpm for 5 min and diluted in PBS and affinity purified with immobilized Protein G. And all mice were euthanized by decapitation at the 4th week, when they cannot generate much more ascites.

### 2.6. Specificity Identification of the AT1-mAb

(1) The purified antibodies were resolved on a 15% sodium dodecyl sulfate-polyacrylamide gel electrophoresis (SDS-PAGE) gel, and the gel was stained with Coomassie Brilliant Blue. (2) The specificity of the antibodies purified from the hybridoma culture supernatant and mouse ascites was determined by BA-ELISA [11], with the wavelength of detection set at 405 nm to provide the optical density (OD) (Spectra Max Plus; Molecular Devices, Sunnyvale, CA): *P*/*N* = (positive OD − blank OD)/(negative OD − blank OD); *P*/*N* > 2.1 was identified as the positive sample; *P*/*N* < 1.5 was identified as the negative sample. The procedure is as follows: 1 *μ*g/mL AT_1_R-ECII peptide dissolved in Na_2_CO_3_ solution (0.1 mol/L, pH 11.0) was coated in 96-well microtiter plates and incubated overnight at 4°C. The wells were saturated with 1% PMT buffer (1% (w/v) bovine serum albumin, 0.1% (v/v) Tween 20 in phosphate-buffered saline (PBS-T), pH 7.4) at 37°C for 1 h. After washing the plates with PBS-T for 3 times, 5 *μ*L samples diluted in 45 *μ*L PMT were added to the plates and incubated at 37°C for 1.5 h. After 3 times' washing, biotinylated rabbit anti-mouse IgG antibodies and biotinylated goat anti-human IgG antibodies (1 : 4500 dilutions in PMT; Zhong Shan Jin Qiao, Beijing, China) were added to the wells and incubated at 37°C for 1 h. After 3 times' washing, the streptavidin-peroxidase conjugate (1 : 3000, Vector, CA) was added to the wells and incubated at 37°C for 1 h. Finally, 2,2-azino-di(3-ethylbenzothiazoline) sulfonic acid- (ABTS-) H_2_O_2_ (Roche, Basel, Switzerland) substrate buffer was applied and reacted in the dark at 37°C for 0.5 h.

### 2.7. The Biological Activity and Functional Identification of AT1-mAb

(1) For the cultivation of primary neonatal rat myocardial cells, we rapidly removed hearts from 0–3-day-old newborn Wistar rats into PBS, cut them into pieces, and washed away the blood cells for 2-3 times with PBS. 35 mg trypsin and 25 mg collagenase type II were dissolved in 30 mL PBS. These digestive enzymes were filtered by 0.22 *μ*m filters and then stand at 37°C. The heart tissue was added into 5 mL beaker and digested in 3 mL enzyme solution for 3–5 min with sustained shaking at 37°C. The supernatant was aspirated into low-glucose Dulbecco's Modified Eagle's medium (DMEM) with 10% fetal bovine serum to terminate digestion. Finally, the cells were centrifuged through a 200-mesh filter and centrifuged at 1000 rpm for 5 min and placed in 10 cm dish. After 2 hours, the supernatant was aspirated and packed in 6-well plates, the adherent cells were fibroblast cells. After culturing for 5 days, the beat of the myocardial cells was observed under a microscope. The AT_1_R blocker valsartan (Sigma, Y0001132) and AT_2_R blocker PD123319 (Sigma, P186) were added to the myocardial cell culture medium. After 10 minutes, purified AT1-mAb was added to the culture medium, and the beat frequency of the myocardial cells was recorded. (2) For isolated vascular ring technology [12], the sodium pentobarbital was used for surgical anesthesia before isolating aortic rings from the mice. The mice were euthanized by decapitation after thoracic aortas were isolated immediately, and a 2 mm vascular ring was removed and affixed to an internal System-610M Multi Myograph bath sensor (Danish Myo Technology A/S Inc., Denmark). The bath contained 5 mL hydroxyethyl piperazine ethanesulfonic acid 4-(2-hydroxyethyl)piperazine-1-ethanesulfonic acid (HEPES; 144 mM NaCl, 5.8 mM KCl, 1.2 mM MgCl_2_N_6_H_2_O, 2.5 mM CaCl_2_, 11.1 mM glucose, and 5 mM HEPES) solution (pH, 7.38–7.40) into which a 95% O_2_/5% CO_2_ gas mixture was continually bubbled. We allowed a 1 h equilibration period before the start of the experiment, and the HEPES solution was replaced every 15 min. Using the vascular ring transducer system, vascular ring tension changes were collected and recorded with LabChart 7 software. The initial passive vascular tension was determined by vascular standardization. HEPES buffer containing 60 mM potassium (144 mM NaCl, 60 mM KCl, 1.2 mM MgCl_2_N_6_H_2_O, 2.5 mM CaCl_2_, 11.1 mM glucose, and 5 mM HEPES; pH, 7.38–7.40) was used for a precontracted vasoactive test. The contractile responses of the vascular rings (tension) to different drugs were defined as a percentage of average contractile intensity to KCl (KCl% = detected drugs/ΔKCl × 100%). (3) The tail-cuff method was used to measure blood pressure in mice, and the position of the mouse was fixed by using a mouse holder maintained on a 37°C hot plate, while a small mouse tail blood flow sensor was placed at the mouse tail root; we then waited until the heart rate stabilized at approximately 375 beats/min. After the blood began to flow smoothly and steadily, we measured systolic blood pressure five times per animal using software of noninvasive automated sphygmomanometer (Japan) and recorded the mean overall blood pressure.

### 2.8. Statistical Analysis

Using SPSS 16.0 (Chicago, IL, USA) for statistical analysis, between-group comparisons were performed with *t*-tests, and comparisons among groups were performed with one-way ANOVA, followed by Tukey's post hoc test (^*∗*^ *P* < 0.05, ^*∗∗*^ *P* < 0.01). All of the bar graphs are shown as the mean ± standard error of the mean (SEM).

## 3. Results

### 3.1. Concentration of AT1-mAb Is Higher in Mouse Ascites Than in Hybridoma Supernatant

**Figure 1:**
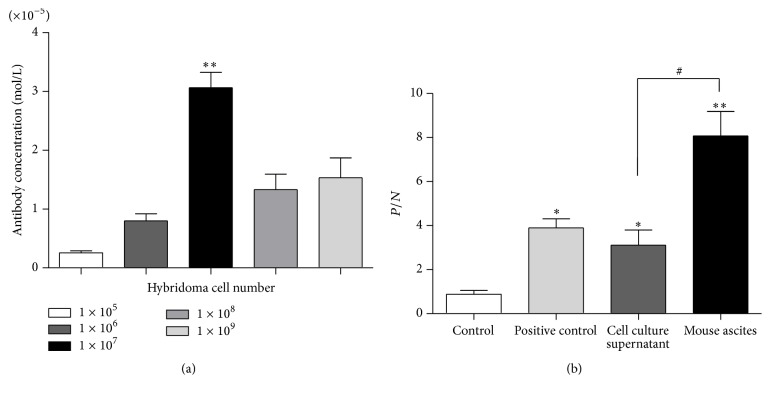
Concentration of AT1-mAb purified from mouse ascites was higher than that from hybridoma supernatant. (a) Concentration of monoclonal antibody that was purified from mouse ascites (^*∗∗*^ *P* < 0.01 versus 1 × 10^−5^, 1 × 10^−6^, 1 × 10^−8^, and 1 × 10^−9^, *n* = 8). (b) Content of AT1-mAb that was purified from the cellular supernatant of hybridomas compared with that from mouse ascites, and positive control was the AT1-AA purified from preeclampsia sera. ^#^ *P* < 0.05 versus cellular supernatant; ^*∗*^ *P* < 0.05 versus control; ^*∗∗*^ *P* < 0.01 versus control. Data represent the mean ± SEM; *n* = 8.

Hybridoma supernatant was collected after 3 days of culture. The results of the bicinchoninic acid (BCA) assay revealed that the concentration of the monoclonal antibodies purified from the supernatant was 3.75 × 10^−6^ mol/L. Different amounts of hybridomas were injected into the abdominal cavity of mice (1 × 10^5^, 1 × 10^6^, 1 × 10^7^, 1 × 10^8^, and 1 × 10^9^), and different concentrations (2.5 × 10^−6^, 9.3 × 10^−6^, 3 × 10^−5^, 1.32 × 10^−5^, and 1.49 × 10^−5^ mol/L) of monoclonal antibodies were obtained. The highest concentration of monoclonal antibodies purified from mouse ascites was from the group that was injected with 1 × 10^7^ hybridomas (*P* < 0.01 versus 1 × 10^−5^, 1 × 10^−6^, 1 × 10^−8^, and 1 × 10^−9^; *n* = 8) (Figure 1(a)). The BA-ELISA results showed that the monoclonal antibodies purified from hybridoma supernatants and mouse ascites were AT1-mAb, AT1-AA purified from preeclampsia sera was used as positive control, and the sera from AT1-AA-negative Balb/C mice were used as control (*P*/*N* values: positive control, 3.96 ± 1.9 versus 0.88 ± 0.43 for controls; *P* < 0.05; hybridoma cell culture supernatant, 3.11 ± 1.81 versus 0.88 ± 0.43 for controls; *P* < 0.05; mouse ascites, 8.07 ± 3.32 versus 0.88 ± 0.43 for controls; *P* < 0.01; *n* = 8); the content of AT1-mAb purified from mouse ascites was higher than that purified from the hybridoma supernatant (*P*/*N*: 8.07 ± 3.12 versus 3.11 ± 1.81 for the supernatant; *P* < 0.05; *n* = 8) (Figure 1(b)) and we extracted the whole protein from the neonatal rat cardiomyocytes, which express the AT_1_R; the Western blot showed that both AT1-AA and AT1-mAb can recognize AT_1_R at the same location specifically (Supplementary Figure 2).

### 3.2. AT1-mAb Purified from Mouse Ascites Belongs to the IgG Immunoglobulin Class

**Figure 2:**
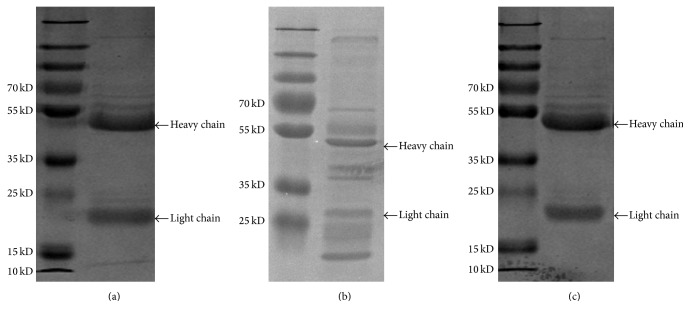
Purity of AT1-mAb from hybridoma supernatant and mouse ascites. (a) IgG control; (b) the antibody extracted from hybridoma supernatant; (c) the antibody extracted from mouse ascites.

SDS-PAGE was used to analyze the purity of hybridoma supernatant and mouse ascites. The results showed that the antibody extracted from hybridoma supernatant showed that there were several other bands in addition to the heavy and light chain (Figure 2(b)); compared to IgG control (Figure 2(a)), the heavy and light chain of purified AT1-mAb were 55 kDa and 25 kDa (Figure 2(c)), which revealed that the antibody extracted from mouse ascites belongs to the IgG immunoglobulin class.

### 3.3. AT1-mAb Purified from Mouse Ascites Increases the Beat Frequency of Newborn Rat Cardiomyocytes

**Figure 3:**
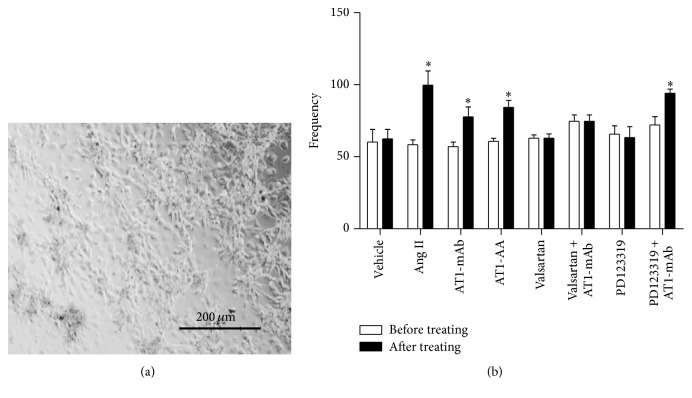
Biological activity of AT1-mAb was identified by changes in beat frequency of neonatal rat myocardial cells. (a) Cultured neonatal rat myocardial cells. Scale bar = 200 *μ*m. (b) Different processing factors had different effects on beating frequency of cultured neonatal rat myocardial cells* in vitro*. ^*∗*^ *P* < 0.05 versus before treating. Data represent the mean ± SEM; *n* = 6.

As shown in Figure 3 (the picture of the newborn rat cardiomyocytes, Figure 3(a)), when 10^−8^ mol/L AT1-mAb (purified from mouse ascites) was added to the culture medium, the beat frequency of the newborn rat cardiomyocytes increased, similar to the effect caused by AT1-AA (10^−8^ mol/L) extracted from patients' sera (AT1-mAb, 77.67 ± 19.67 versus 57.08 ± 5.29 before treatment; AT1-AA, 84.33 ± 8.33 versus 60.67 ± 3.79 before treatment; *P* < 0.05; *n* = 6); Ang II (10^−7^ mol/L), the endogenous AT_1_R agonist, also increased the beat frequency of the rat cardiomyocytes (99.71 ± 22.14 versus 58.36 ± 5.75 before treatment; *P* < 0.05; *n* = 6); AT_1_R blocker valsartan (10^−7^ mol/L) could block the beating frequency increase caused by AT1-mAb, although the AT_2_R blocker PD123319 (10^−7^ mol/L) did not have an inhibitory effect (94.08 ± 2.89 versus 72.03 ± 5.77 before treatment; *P* < 0.05; *n* = 6) (Figure 3(b)). These data reveal that AT1-mAb can increase the beat frequency of newborn rat cardiomyocytes by activating the AT_1_R, but not AT_2_R.

### 3.4. AT1-mAb Purified from Mouse Ascites Induces Contraction of the Thoracic Aorta

**Figure 4:**
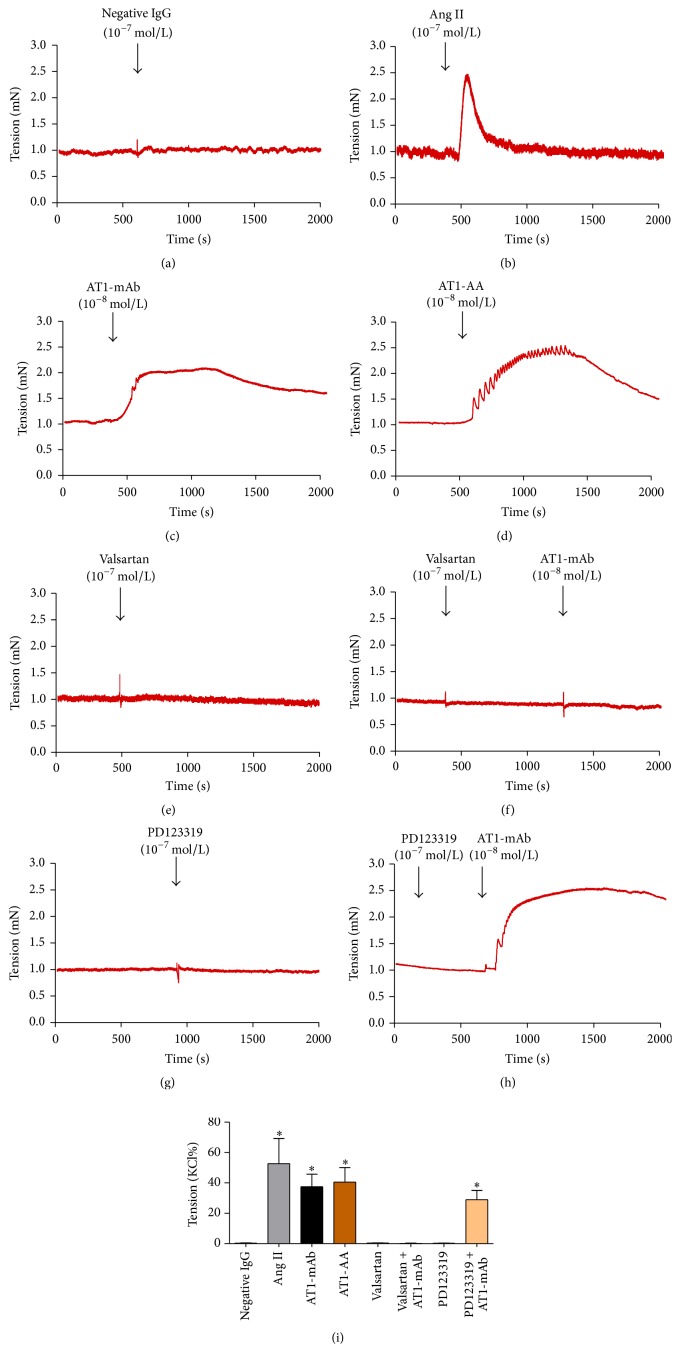
Biological activity of AT1-mAb was identified by isolated thoracic aorta ring technology. (a) Negative IgG isolated from healthy mouse serum; (b) Agonist of AT_1_R, Ang II; (c) AT1-mAb; (d) for AT1-AA exert the same effect on the contractile force of mouse thoracic aorta; (e) blocking agent for AT_1_R, valsartan; (f) valsartan + AT1-mAb; (g) blocking agent for AT_2_R, PD123319; (h) PD123319 + AT1-mAb; and (i) statistical graph of isolated vascular ring. ^*∗*^ *P* < 0.05 versus negative IgG. Data represent the mean ± SEM; *n* = 6.

The results in Figure 4 show that both 10^−7^ mol/L Ang II and 10^−8^ mol/L AT1-mAb purified from mouse ascites could induce vasoconstriction. Compared to negative IgG group (Figure 4(a)), the effect of AT1-mAb on vascular function was the same as that caused by AT1-AA (10^−8^ mol/L) extracted from patients' sera (tension values: Ang II, Figure 4(b), 52.71%  ±  20.86% versus 3.72%  ±  2.26% for negative IgG; AT1-mAb, Figure 4(c), 37.51%  ±  16.42% versus 3.72%  ±  2.26% for negative IgG; AT1-AA, Figure 4(f), 40.50%  ±  19.20% versus 3.72%  ±  2.26% for negative IgG; *P* < 0.05; *n* = 6). When valsartan (10^−7^ mol/L) was added first, the vasoconstriction caused by AT1-mAb was blocked (Figure 4(d), *P* > 0.05; *n* = 6). However, when PD123319 (10^−7^ mol/L) was added first, the vasoconstriction caused by AT1-mAb could not be blocked (Figure 4(e), 28.93%  ±  10.04% versus 3.72%  ±  2.26% for negative IgG; *P* < 0.05; *n* = 6). Statistical graph of isolated vascular ring is Figure 4(g). These results demonstrate that AT1-mAb exerts its biological functions by activating the AT_1_R, but not the AT_2_R.

### 3.5. AT1-mAb Directly Increases the Blood Pressure of Pregnant Mice

**Figure 5:**
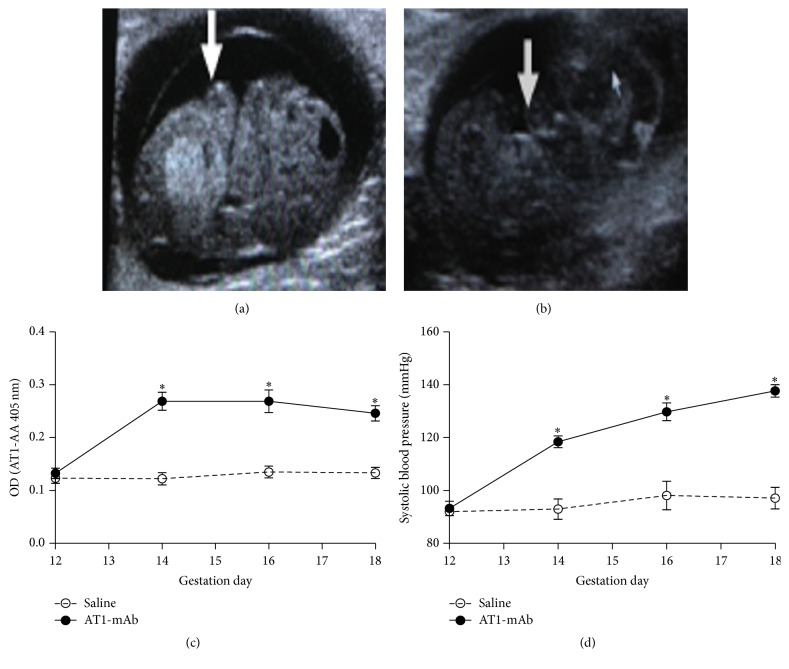
Elevated blood pressure of pregnant mice caused by AT1-mAb. (a) Control group; (b) AT1-mAb group; (c) content of AT1-mAb in sera of mice; (d) systolic blood pressure levels in the pregnant mouse model. ^*∗*^ *P* < 0.05 versus saline. Data represent the mean ± SEM; *n* = 6.

Twelve-week-old healthy adult females and males were caged together, and ultrasonography showed successful pregnancy. On the 13th day of gestation, AT1-mAb (20 *μ*g/g) [13] was injected into the pregnant mice through the tail vein, and an equal dose of normal saline was injected into the mice in the control group (control group, Figure 5(a); AT1-mAb group, Figure 5(b)). The content of the AT1-mAb in pregnant mice sera was tested on days 14, 16, and 18 of gestation. The levels of AT1-mAb began to rise from the 14th day of gestation and were maintained to the 18th day (OD on day 14 of gestation: 0.27 ± 0.05 versus 0.12 ± 0.02 for saline; day 16 of gestation, 0.26 ± 0.05 versus 0.14 ± 0.016 for saline; day 18 of gestation, 0.25 ± 0.03 versus 0.13 ± 0.02 for saline; *P* < 0.05; *n* = 6; Figure 5(c)). On alternate days, blood pressures were measured using mouse tail sensors. The systolic blood pressures of AT1-mAb-positive pregnant mice were significantly higher than those of mice in the control group on days 14, 16, and 18 of gestation (118.46 ± 5.82 versus 92.94 ± 7.64 mmHg for saline on day 14 of gestation: 129.78 ± 8.92 versus 98.08 ± 10.81 for saline on day 16 of gestation; and 137.69 ± 4.73 versus 97.10 ± 9.17 for saline on day 18 of gestation; *P* < 0.05; *n* = 6; Figure 5(d)).

## 4. Discussion

A large number of studies have identified AT1-AA as a harmful factor that exists in several diseases associated with cardiovascular disturbances, especially in patients with preeclampsia [14]. It plays an agonistic role by specifically interacting with AT_1_R-ECII. However, other unknown pathological mechanisms of AT1-AA action need to be further explored. Obtaining AT1-AA is the primary goal in establishing AT1-AA-positive animal model and studying the pathological significance of and mechanisms underlying this antibody in diseases such as preeclampsia [15]. Currently, isolating antibody from the sera of preeclamptic patients is a basic approach to obtaining AT1-AA; however, it is crucial to acquire other sources of AT1-AA because of the limited availability of clinical patients' sera, collection difficulties, and large losses when purification is not timely [16]. Active immunization is an alternate method for obtaining AT1-AA, which involve mixing synthetic AT_1_R-ECII with incomplete Freund's adjuvant to retrieve the antigen and injecting it into the animals by subcutaneous injection with a booster injection administered at 2-week intervals. Typically, it is possible to procure high titer until the 8th week [17]. Therefore, active immunization requires a relatively long time and a large dose of antigen peptides. In addition, the retrieved antibodies are usually polyclonal in nature. Therefore, it is important to obtain AT1-mAb with high potency and specificity to allow the study of the pathologic roles of AT1-AA [18]. Monoclonal antibody technology was used in the present experiment. The synthetic peptide AT_1_R-ECII was used to actively immunize Balb/C mice through subcutaneous injection, and then single B lymphocytes capable of secreting AT1-mAb were hybridized with unlimited proliferating myeloma cells to obtain hybrid cells that could be immortalized and secrete AT1-mAb. The hybridomas were selected using ELISA, and AT1-mAb was extracted from mouse ascites after hybridomas were inoculated into Balb/C mice for 2 weeks. Our results showed that AT1-mAb can be extracted from hybridoma supernatants and mouse ascites, the content of AT1-mAb purified from mouse ascites was higher than that purified from the hybridoma supernatant, and the AT1-mAb purified from mouse ascites belongs to the IgG immunoglobulin class. Additionally, compared to extracting AT1-mAb from hybridoma supernatants, extracting AT1-mAb from mouse ascites is of higher potency and less cost. Therefore, we chose the mouse ascites method for preparing purified AT1-mAb.

Numerous studies have shown that there are a variety of G-protein-coupled receptors, including AT_1_R, on the surface of mouse vascular endothelial cells [19] and smooth muscle cells [20], as well as on neonatal rat cardiomyocytes [21]. Ang II, an endogenous agonist of the AT_1_R, binds to AT_1_R and causes a positive inotropic effect, such as the increased beat frequency of cardiomyocytes and induction of vasoconstriction [22]. Therefore, after the specificity of the AT1-mAb purified from mouse ascites was confirmed, neonatal rat cardiomyocyte experiments and isolated vascular ring technology were used to evaluate the biological activity of AT1-mAb. When AT1-mAb was added to the culture medium of neonatal rat cardiomyocytes, the beat frequencies of cardiomyocytes were significantly increased, similar to the effect of AT1-AA isolated from patients' sera. When the AT_1_R blocker valsartan was added to the medium first, the cell surface receptors were blocked by blocking the effect of AT1-mAb on beat frequency in rat neonatal cardiomyocytes; the AT_2_R blocker PD123319 did not have a blocking effect. Furthermore, similar to AT1-AA purified from patients' sera, AT1-mAb purified from mouse ascites caused observable vasoconstriction in mouse thoracic aorta. If the AT_1_R blocker valsartan was added prior to giving AT1-mAb treatment in the isolated vascular ring, vasoconstriction was completely blocked, whereas the AT_2_R blocker PD123319 did not block the effect. All of the above observations demonstrate that AT1-mAb purified from mouse ascites does not bind to AT_2_R but rather specifically activates AT_1_R, thereby exerting its agonist-like biological effects. In addition, compared to the instantaneous vasoconstrictor effect caused by Ang II, AT1-mAb and AT1-AA caused continued contraction in mouse thoracic aorta, which suggests that activation of AT_1_R without desensitization may be an important mechanism whereby AT1-AA produces pathological and damaging effects. Additional studies are required to further explore this putative mechanism.

Finally, to confirm that AT1-mAb plays a biological role* in vivo*, we injected AT1-mAb into Balb/C mice* via* the tail vein on the 13th day of gestation. Researchers demonstrated that the half-life of clinically sourced AT1-AA is about 10 days, so that the AT1-AA can be retained in the body for a long period, and thus mouse blood pressure may also be maintained at a high level after AT1-AA injection [23]. In addition, when pregnant mice were given an injection of AT1-mAb purified from ascites, blood pressure on day 14 of gestation was increased and continued until the end of the experiment. This suggests that AT1-mAb extracted from ascites simulated patient-derived antibodies both* in vitro* and* in vivo*.

**Figure 6:**
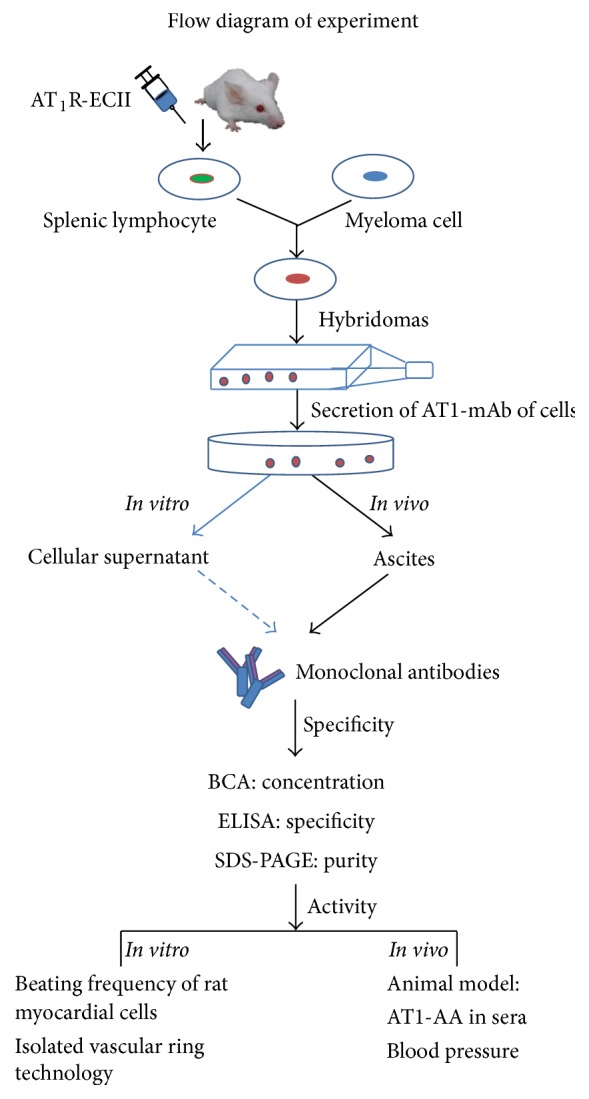
Flowchart of experimental procedures.

In summary, we prepared AT1-mAb with high specificity, high concentration, and biological activity by inoculating hybridomas that secrete AT1-mAb into the mouse abdominal cavity (Figure 6). Preparation of AT1-mAb is required to establish AT1-AA-positive animal models and will be a useful tool for the research of clinical diseases that manifest a high titer of AT1-AA, such as preeclampsia.

## Supplementary Material

Supplementary Figure 1: The proteins sequence of the angiotensin II type 1 receptor of the Homo sapiens and Mus musculus were compared by using Basic Local Alignment Search Tool, the murine and human AT1R-ECII share 92% homology.
Supplementary Figure 2: Both AT1-AA from preeclampsia serum and AT1-mAb can recognize AT1R at the same location in Western blot.

## Figures and Tables

**Table 1:** Clinical data of patients with preeclampsia subjects.

Patients	Preeclampsia (*n* = 6)
Maternal age (years)	30 (27–34)
Gestational age at sampling (weeks)	37.5 (35–40)
Ethnic background	Han
SBP (mmHg)	152 ± 11
DBP (mmHg)	105 ± 6
Proteinuria (mg/day)	478 ± 33.5

SBP, systolic blood pressure; DBP, diastolic blood pressure.
