# Supplementary material for: Preparation and Biological Activity of the Monoclonal Antibody against the Second Extracellular Loop of the Angiotensin II Type 1 Receptor
Source: J Immunol Res. 2016 Jan 20;2016:1858252. doi: 10.1155/2016/1858252 (PMC4745622; doi:10.1155/2016/1858252)

Supplementary Figure1

A

Mus musculus angiotensin II receptor, type 1a

MALNSSTEDGIKRIQDDCPRAGRHSYIFVMIPTLYSIIFVVGIFGNSLVVIVIYFY
MKLKTVASVFLLNLALADLCFLLTLPLWAVYTAMEYRWPFGNHLCKIASASV
SFNLYASVFLLTCLSIDRYLAIVHPMKSRLRRTMLVAKVTCIIIWLMAGLASLPA
VIHRNVYFIENTNITVCAFHYESRNSTLPIGLGLTKNILGFLFPFLIILTSYTLIWK
ALKKAYEIQKNKPRNDDIFRIIMAIVLFFFFSWVPHQIFTFLDVLIQLGVIHDCK
IADIVDTAMPITICIAYFNNCLNPLFYGFLGKKFKKYFLQLLKYIPPKAKSHSSL
STKMSTLSYRPSDNMSSAAKKPASCSEVE

B

Homo sapiens angiotensin II receptor, type 1

MILNSSTEDGIKRIQDDCPKAGRHNYIFVMIPTLYSIIFVVGIFGNSLVVIVIYFY
MKLKTVASVFLLNLALADLCFLLTLPLWAVYTAMEYRWPFGNYLCKIASASV
SFNLYASVFLLTCLSIDRYLAIVHPMKSRLRRTMLVAKVTCIIIWLLAGLASLPAI
IHRNVFFIENTNITVCAFHYESQNSTLPIGLGLTKNILGFLFPFLIILTSYTLIWKA
LKKAYEIQKNKPRNDDIFKIIMAIVLFFFFSWIPHQIFTFLDVLIQLGIIRDCRIAD
IVDTAMPITICIAYFNNCLNPLFYGFLGKKFKRYFLQLLKYIPPKAKSHSNLSTK
MSTLSYRPSDNVSSSTKKPAPCFEVE

C

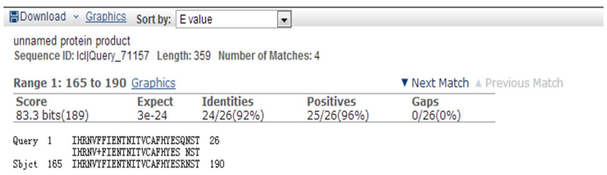

Supplementary Figure 2

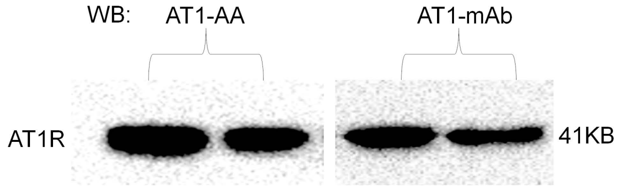

Supplement: Supplementary file 1 — Supplementary Figure 1: The proteins sequence of the angiotensin II type 1 receptor of the Homo sapiens and Mus musculus were compared by using Basic Local Alignment Search Tool, the murine and human AT1R-ECII share 92% homology. Supplementary Figure 2: Both AT1-AA from preeclampsia serum and AT1-mAb can recognize AT1R at the same location in Western blot. [file 1858252.f1.pdf]
